# Nonlinear Network Reconstruction from Gene Expression Data Using Marginal Dependencies Measured by DCOL

**DOI:** 10.1371/journal.pone.0158247

**Published:** 2016-07-05

**Authors:** Haodong Liu, Peng Li, Mengyao Zhu, Xiaofei Wang, Jianwei Lu, Tianwei Yu

**Affiliations:** 1 School of Software Engineering, Tongji University, Shanghai, China; 2 School of Life Sciences and Technology, Tongji University, Shanghai, China; 3 College of Information Science and Engineering, Shandong University of Science and Technology, Shandong, China; 4 Institute of Translational Medicine, Tongji University, Shanghai, China; 5 Department of Biostatistics and Bioinformatics, Rollins School of Public Health, Emory University, Atlanta, Georgia, United States of America; University of California, Los Angeles, UNITED STATES

## Abstract

Reconstruction of networks from high-throughput expression data is an important tool to identify new regulatory relations. Given that nonlinear and complex relations exist between biological units, methods that can utilize nonlinear dependencies may yield insights that are not provided by methods using linear associations alone. We have previously developed a distance to measure predictive nonlinear relations, the Distance based on Conditional Ordered List (DCOL), which is sensitive and computationally efficient on large matrices. In this study, we explore the utility of DCOL in the reconstruction of networks, by combining it with local false discovery rate (lfdr)–based inference. We demonstrate in simulations that the new method named nlnet is effective in recovering hidden nonlinear modules. We also demonstrate its utility using a single cell RNA seq dataset. The method is available as an R package at https://cran.r-project.org/web/packages/nlnet.

## Introduction

High-throughput expression techniques such as microarray, deep sequencing, and liquid chromatography-mass spectrometry (LC-MS) are able to generate measurements of tens of thousands of biological units simultaneously [1, 2]. Identifying relations between the biological units and detecting modules in the system can result in a better understanding of the underlying regulatory system [3–5]. However, the large number of biological units involved, the complexity of their associations, and the under-determined nature of the problem poses great challenges toward data analysis [6].

The topic of network reconstruction from high-throughput data has received a great deal of attention in recent years. A number of methods with different objectives have been proposed, including network reconstruction based on marginal correlation [7, 8], Gaussian graphical models [9, 10], Bayesian network [11, 12], mutual information-based network inference [13–16], *etc*. Some methods go beyond inference using a single approach, by taking previous knowledge into consideration [17], or combining the predictions from multiple methods [18, 19]. Given a gene expression dataset, different algorithms put emphasis on different aspects of data characteristics, and the prediction of different algorithms always show large discrepancies [20].

Nonlinear and complex relations between genes has been widely documented. They may represent nonlinear response, conditional dependency, or time-lagged dependency [21–24]. Mutual information-based network reconstruction methods can establish links between genes based on nonlinear relations [13–16, 25]. However nonlinear relations can be diverse, and generally the statistical power of detecting such relations is lower compared to detecting linear relations based on correlation.

We have previously developed a very sensitive method to detect predictive nonlinear relations, named the Distance Based On Conditional Ordered List (DCOL) [26]. Given the method is rank-based, and the asymptotic normality of the test statistic under the null hypothesis, the computation is very efficient on large matrices. Also, it is amenable to statistical inference, i.e. rigorously defining links between genes based on p-value or false discovery rate [27]. These properties make DCOL a very good candidate for nonlinear network reconstruction.

**Fig 1:**
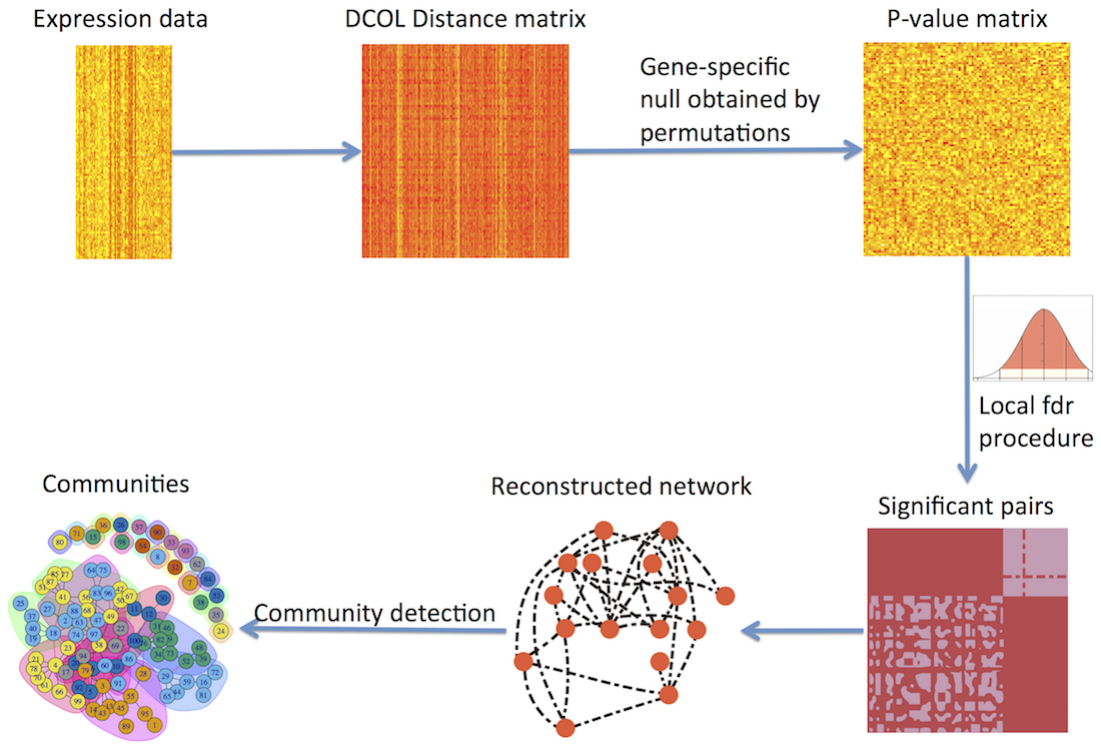
The overall workflow of the method.

In this study, we develop a network reconstruction method based on DCOL. First, we compute pairwise relations between genes using DCOL. We then conduct gene-wise false discovery rate inference [27] to determine significant links for each gene. Such an approach will yield a network with marginal nonlinear dependencies, i.e. not considering conditional dependencies. It has been shown previously that reconstruction based on marginal linear dependencies performed very well compared to other methods [20]. Given that nonlinear relations can be quite diverse, and the statistical power of detecting different types of nonlinear relations can vary substantially especially when considering conditional dependencies, it is our belief that constructing a network using marginal dependencies and allowing users to trim the links based on biological considerations is a viable approach.

we demonstrate the performance of our method using simulations, mostly in its capability of recovering hidden community structures, in comparison with a few other methods. We also apply the method to a real dataset. The real data analysis shows that our method successfully detects meaningful biological relations supported by existing knowledge, as well as detects plausible new links. In the following discussions, we refer to our method as nlnet. Fig 1 shows the general workflow of the method.

## Methods

### Distance Based on Conditional Ordered List (DCOL)

Given a data matrix *G*_*p*×*n*_ with *p* genes from *n* samples, the key issue is to find significant relations between genes that are either linearly or nonlinearly associated. We have previously proposed the Distance Based on Conditional Ordered List (DCOL) as a non-linear distance [26]. To find the *DCOL*(*g*_*j*_|*g*_*i*_),∀*j* = 1,…,*p*, where *g*_*i*_ is a row-vector in matrix *G*, we sort the columns of the matrix based on the value of *g*_*i*_ first to create an ascending order, $\left\{G^{*}:\;g_{i,1}^{*}\leq g_{i,2}^{*}\leq \ldots \;\leq \;g_{i,n}^{*}\right\}$. Then we obtain the DCOL based on the sorted data,

$$DCOL\left(g_{j}|g_{i}\right),\;=\;\frac{1}{\left(n-1\right)}\overset{n}{\underset{k\;=\;2}{\sum }}\left|g_{j,k}^{\text{*}}-g_{j,k-1}^{\text{*}}\right|,\;\forall j\;=\;1,\ldots ,p$$

In actual computation, for each *g*_*i*_, we permute the columns of matrix *G* based on the order of the values of *g*_*i*_, and find *DCOL*(*g*_*j*_|*g*_*i*_) for all *j’s* simultaneously.

### Null Distribution

It has been shown that the DCOL under the null hypothesis, i.e. the two genes are independent of each other, follows normal distribution [26]. We can obtain a gene-specific null distribution by permutation. In order to estimate a null distribution of genes, we randomly permute the columns of data matrix *G* for B times (B = 500 in this study). For each permutation, we calculate the sum of absolute difference between adjacent columns in a row,

$${DCOL}^{\left(b\right)}(g_{j}|null)\;=\;\frac{1}{\left(n-1\right)}\sum_{k\;=\;2}^{n}\left|g_{j,k}^{\left(b\right)}-g_{j,k-1}^{\left(b\right)}\right|,\;\forall j\;=\;1,\ldots ,p$$

We then estimate the null distribution parameters for gene *i*, $m_{i}\;=\;\frac{1}{B}\sum_{b\;=\;1}^{B}{DCOL}^{\left(b\right)}(g_{j}|null)$ and $s_{i}^{2}\;=\;\frac{1}{B}\sum_{b\;=\;1}^{B}{\left({DCOL}^{\left(b\right)}(g_{j}|null)-m_{i}\right)}^{2},\forall j\;=\;1,\ldots ,p$.

### Gene Network Reconstruction

By applying the DCOL we introduced in section 2.1, we find the gene distance matrix *D*_*p*×*p*_, where *D*_*i*,*j*_ = *DCOL(g*_*j*_|*g*_*i*_). For each column of the matrix, we compare the distance vector to the null distribution to obtain the one-sided p-value, $P_{i,j}\;=\;\Phi^{-1}(\frac{D_{i,j}-m_{j}}{s_{j}})$. We then feed the column vector of p-values to the fdrtool package [28] to obtain local false discovery rate (lfdr) values [29], which is the posterior probability that *g*_*j*_ is dependent on *g*_*i*_ Up to this step, we have a matrix *L*_*p*×*p*_ of local fdr values.

Next we threshold the local fdr values to obtain connections between genes. In order to control the overall level of edge density in the network, we apply a dynamic thresholding procedure. A parameter is provided to set the target average degrees over all nodes. This target corresponds to a target fdr value. If this target fdr is between 0.05 and 0.2, we will use the target fdr as cutoff. Otherwise, if the target fdr is larger than 0.2, we will use 0.2; if the target fdr is smaller than 0.05, we will use 0.05 as cutoff.

### Community Detection in Network

As the biological system is modular [30], to facilitate data interpretation, we further decompose the gene network into communities. There are many mature community detection methods which are commonly applied in social computing but are also suitable for genes networks. In this study, we apply two common community finding methods: multi-level optimization of modularity, and label propagation [31, 32].

### Simulation

We conduct a simulation study to examine the capability of the proposed method to recover nonlinear gene modules. The reason for focusing on module recovery, rather than link recovery, is because in the nonlinear situation, methods have different statistical power on different types of nonlinear functions, and it is unreliable to define conditional dependency given the existence of measurement noise. As modules are more robust against the gain/loss of a small proportion of links, we believe the recovery of gene modules is a more meaningful goal.

We conduct the simulation by setting parameters in two main scenarios. We set the average number of genes in a module to 100, and set the number of hidden modules to 10 or 20. An additional 10% pure noise genes are also added. In each scenario, the number of genes are calculated based on modules number and size, namely 1100 genes for scenario 1 and 2200 genes for scenario 2. Noise is added to the generated gene expression vectors by adjusting the noise level from 0.2 to 0.8 (noise standard deviation divided by true signal standard deviation). We use three different mechanisms for generating modules, (1) 0-dependent approach, (2) 1-dependent approach, (2) 2-dependent approach. To establish nonlinear relations, we use four link functions including (1) linear function, (2) sine function, (3) box wave function, (4) absolute value function.

For 0-dependent approach, for each module, we generate the expression level of a hidden controlling node ***x*** by sampling the standard normal distribution. Then for each gene in the module, a link function *f*() would be randomly chosen from the function group, and the gene expression level is generated as *y* = *f*(*x*) + *ε*, where *ε* is random noise. For 1-dependent approach, for each module, the first gene is generated by sampling the standard normal distribution. From the second gene on, one existing gene is randomly selected, and a link function is randomly selected. The expressions of the new gene is generated as *y*^(*new*)^ = *f*(*y*^(*selected*)^) + *ε*. The 2-dependent approach is similar to the 1-dependent approach, except each new gene depends on two existing genes, *y*^(*new*)^ = *f*(*y*^(*selected*1)^) + *g*(*y*^(*selected*2)^) + *ε*

## Results and Discussions

### Simulation Result

We compared our algorithm with three methods that deal with nonlinear associations. One is the mutual information-based network inference methodology ARACNE, and its R package name is *parmigene* [14]. Two other methods are nonlinear clustering methods: General Dependency Hierarchical Clustering (GDHC) [26] and the K-profile method [33].

**Fig 2:**
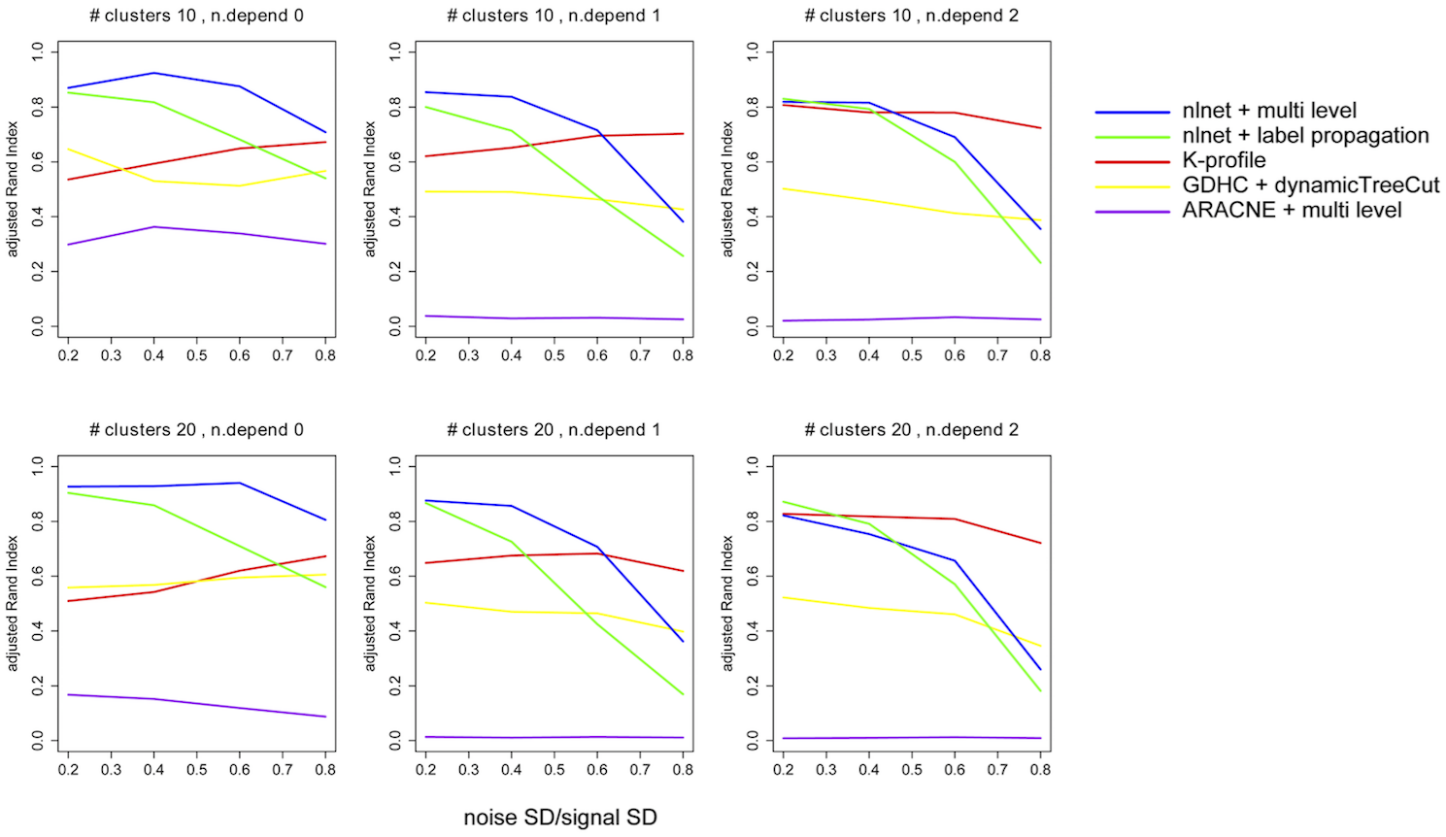
Simulation results on nonlinear community detection.

The Adjusted Rand Index (ARI) [34] is used to evaluate the results. Higher ARI values means better agreement between detected and hidden true modules. The simulation results are shown in Fig 2. There are two rows in the figure. The first row represents algorithm performance under the 10 hidden gene module circumstance, and the second row represents 20 hidden module circumstances.

For the 0-dependent scenarios, apparently the result of nlnet combined with the multi-level algorithm outperformed all other methods across all noise levels. The result by ARACNE is the worst one, in which the optimal ARI is below 0.5 and in most of cases, it stays at the bottom of the plots. So it is safe to say that the ARACNE algorithm is not adapted well to our benchmark.

For the 1-dependent scenarios, nlnet performs the best when noise level is low. The result of K-profile becomes better when the noise level grows higher, while GDHC and ARACNE lag behind. This trend persists into the 2-dependent scenarios. We notice that the 2-dependent scenarios, the dependencies between gene pairs are weaker but more pervasive. When noise level is high, it is likely that network-based methods lose too many true connections, causing the modules to break up.

The second row of Fig 2 (20 true modules) shows a similar trend as the first row (10 true modules). Compared with nlnet paired with multi-level or label-propagation method, the K-Profile algorithm only shows a better result in cases where the noise is high, or the dependencies are weaker but more pervasive. Overall, the nlnet-based module recovery outperforms the existing methods in lower noise situations, and when the dependency structure is simpler.

### Real Data Result

We apply the method on the single-cell RNA seq data on sensory organs of the neonatal inner ear in mice (GSE71982) [35]. In the inner ear, sound and acceleration stimuli are transduced by cochlear and utricular sensory epithelia. We compare the cochlear and utricular single cell RNA seq data through our method of non-linear network reconstruction and community detection. Firstly, we filter the sequencing data by requiring non-zero values of each selected gene to be greater than 2/3 of the samples in either of the tissue types. Secondly we log-transform the data by taking *log*(*x*+1) of all values. Finally, we apply our algorithm to construct networks for utricular and cochlear sensory epithelia separately. For community detection, the minimum module size of is set to 100.

**Fig 3:**
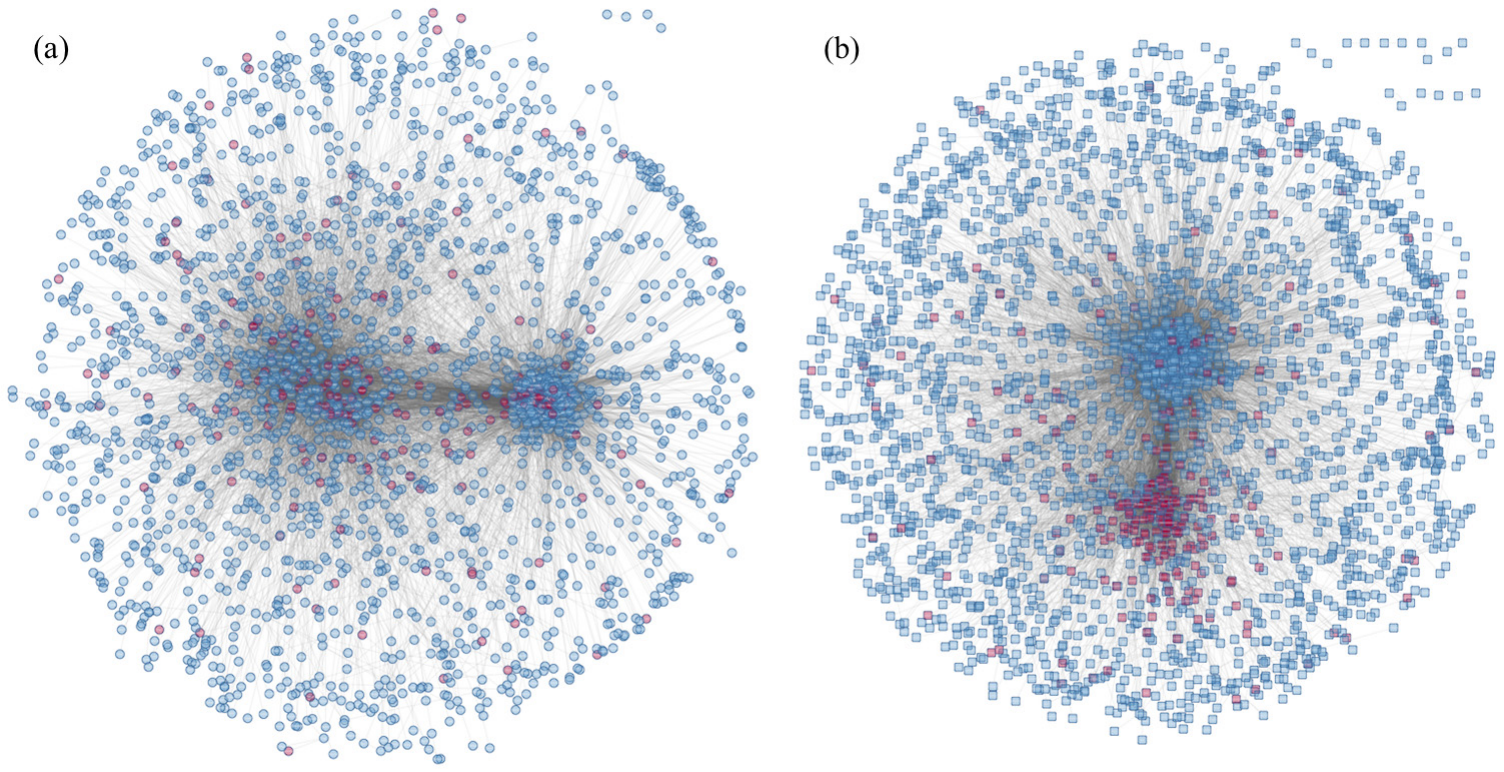
Detected networks in cochlear and utricular cells. (a) The cochlear network; nodes with 50 or more connections in the utricular network are colored red. (b) The utricular network; nodes with 50 or more connections in the cochlear network are colored red.

Fig 3 shows the overall network. The cochlear network has 2412 connected genes and 33846 connections. The utricular network has 2906 connected genes and a total of 45068 connections. With an average degree of 15.5 compared to 14 in the cochlear network, the utricular network is more densely connected. We color the highly connected genes in the utricular network (≥50 connections) red in the cochlear network (Fig 3a), and vice versa (Fig 3b). We see that many highly connected genes in one cell type are no longer highly connected in the other type of cell.

Functional analysis on highly connected genes (≥50 connections in cochlear cells alone, or ≥50 connections in utricular cells alone) shows an over-representation of regulatory and sensory developmental function (Table 1).

**Table 1:** Top 5 GO biological pathways over-represented by highly connected genes in cochlear or utricular cells.

GOBPID	Pvalue	Term
**Cochlear**		
GO:0032259	0.000334	methylation
GO:0001886	0.00224	endothelial cell morphogenesis
GO:0010039	0.00224	response to iron ion
GO:0007600	0.00247	sensory perception
GO:0006400	0.00439	tRNA modification
**Utricular**		
GO:0009888	9.89e−06	tissue development
GO:0007605	1.24e−05	sensory perception of sound
GO:0007275	2.88e−05	multicellular organismal development
GO:0003007	3.01e−05	heart morphogenesis
GO:0048839	3.26e−05	inner ear development

In our result, there are two communities (523 and 1726 genes respectively) in cochlear cells. The second community shows a clear functional link to inner ear and sensory development (Table 2). There are 5 communities (535, 205,159,1235, and 553 genes respectively) in utricular cells. However there is no clear link to inner ear development at the community level. Given the communities are too large to yield simple biological interpretations, we focus on some genes that have been widely studied in ear development.

**Table 2:** Top 5 GO biological pathways over-represented by community 2 of cochlear cells.

GOBPID	Pvalue	Term
GO:0098609	0.000211	cell−cell adhesion
GO:0048839	0.000266	inner ear development
GO:0007423	0.000289	sensory organ development
GO:0098602	0.000702	single organism cell adhesion
GO:0006928	0.000915	movement of cell or subcellular component

**Fig 4:**
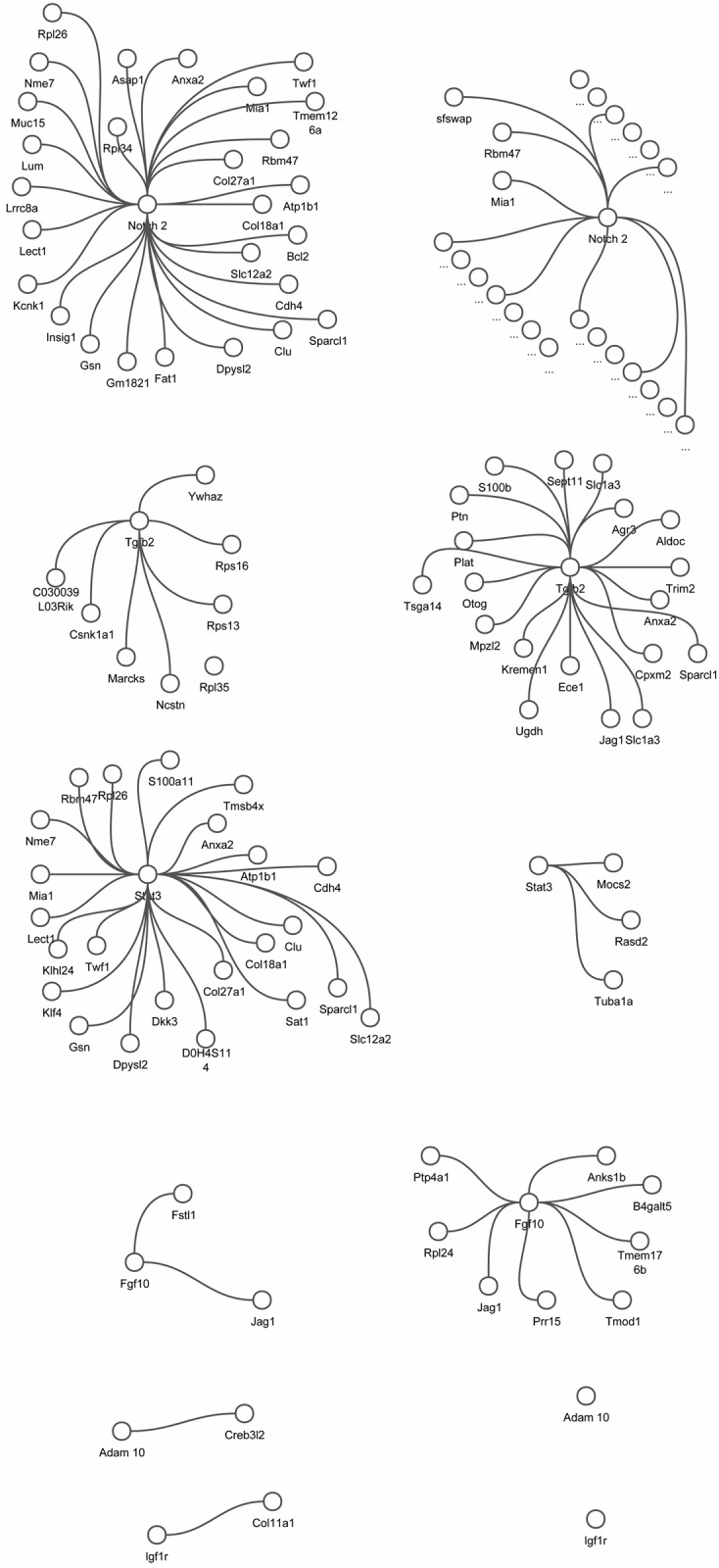
Network surrounding Notch2, Tgfb2, Stat3, Fgf10, Adam10 and Igf1r in cochlear (left column) and utricular (right column) cells.

Genes such as Tgfb2, Stat3, Notch2, and Fgf10 (Fig 4), were widely studied in the development of the ear. These genes are found in communities of both cochlear and utricular system. But their connection patterns are different between the two cell types.

We can find there are more genes connected with Notch2 and Tgfb2 in utricle than cochlea. The Notch signaling pathway is thought to regulate development of inner ear. Overall, Notch2 has 28 connections in the cochlear cells, and 159 connections in the utricular cells. Functional analysis using GOstats [36] shows that Notch2-connected genes over-represent biological processes related to the genesis of cellular structure in cochlear cells, and sensory development in utricular cells (Table 3). Aberrant number and arrangement of hair cells and supporting cells of the ear are caused by mutations in the Notch signaling pathway. Moayedi *et al* found the Sfswap^Tg^ mutation can result in defects in the mouse ear, and the defects were consistent with disrupted Notch signaling [37]. In this study, Sfswap mutants exhibit defects in hair cells and supporting cells in the cochlea, but exhibited much more severe vestibular organ defects. In our results, we can only find the connection between Sfswap and notch pathway in the utricular cells, which is part of the vestibular organ.

**Table 3:** Top 5 GO biological pathways for Notch2-connected genes in cochlear or utricular cells.

GOBPID	Pvalue	Term
**Cochlear**		
GO:0031333	0.000105	negative regulation of protein complex assembly
GO:0090066	0.000208	regulation of anatomical structure size
GO:0001886	0.000251	endothelial cell morphogenesis
GO:0030198	0.000434	extracellular matrix organization
GO:0042989	0.000499	sequestering of actin monomers
**Utricular**		
GO:0007605	2.35E-07	sensory perception of sound
GO:0043583	4.31E-06	ear development
GO:0007600	3.14E-05	sensory perception
GO:0042490	6.58E-05	mechanoreceptor differentiation
GO:0042472	7.38E-05	inner ear morphogenesis

Slowik *et al* found the ligand of Notch pathway, Jag1, can attend mammalian hair cell regeneration through Tgf family [38]. Jag1 is connected with Tgfb2 in cochlear cells, but not in utricular cells, although Tgfb2 is connected with more genes in utricular cells, which over-represent neurological system process. Stat3 has more direct link genes in cochlea than utricle. Qin *et al* found the cross talk of Klf4 and Stat3 can regulates axon regeneration, which was also associated with ear nervous sensor development [39, 40]. In the estimated networks, Klf4 is connected with Stat3 only cochlear cells.

Fgf10 plays a major role in ear morphogenesis. Urness *et al* found a dosage-sensitive requirement for Fgf10 in vestibular development [41], and that the mutant cochlear epithelium of the Fgf10−/− embryos lacks Reissner’s membrane and a large portion of the outer sulcus, consistent with earlier findings on a null mutation in Fgfr2b, one of Fgf10’s main receptors [42]. In our result, Fgf10 is connected with Jag1 in both cochlear and utricular system. Jag1, a notch ligand, is also required for sensory progenitor development in the mammalian inner ear [43]. Together, our result suggests the notch pathway has interacted with other signaling pathways such as the Tgf [38] and Fgf [43] pathways to create sensory organs of ear.

There are some other genes that only have connections in one of the cell types. As an example, Igf1r is a classic transmembrane tyrosine kinase receptor and is believed to mediate all cellular responses to the Igf ligands. During the development of cochlea, Igf signaling and PI3K/AKT signaling can regulate cochlear length and hair cell number. Igf signaling also regulates Atoh expression by PI3K/Akt signaling. PI3K/Akt signaling is considered to be the downstream of Igf signaling [44, 45]. Igf1r is found in a community of cochlear network, with connection to another important gene Chol11a1 in cochlea development [46], but not in the utricular system (Fig 4). Adam10 is also related with the development of cochlear. Yan *et al* found regional expression of the Adam10 in developing chicken cochlea [47]. Lin *et al* also showed that Adam10 is expressed and regulated in the early developing cochlea [48]. Some more genes such as Rbpj, that take part in the functional and morphological development of cochlear organ [49], also only found in the community of cochlear network.

Overall, our results show some agreements and distinctions between the cellular networks of cochlear and utricular cells, much of which agree with existing knowledge, with some new links that are plausible but requires experimental studies to validate. This indicates the nlnet method can produce biologically meaningful results.

The computation of the nlnet method is relatively efficient. As shown in Table 4, among all the methods used in simulation, nlnet is the second fastest. It uses ~20 seconds for a data matrix of dimension 1100 × 100, on a laptop computer with a 2.5 GHz intel i7 processor. When the data dimension is larger in real data analysis, nlnet is the fastest among all methods tested, finishing in ~5 minutes for a data matrix of dimension 3724 × 164.

**Table 4:** Computing time.

Algorithms	CPU time (seconds)*
Simulations^#^ (1100 genes, 100 samples)	
ARACNE+ multi level	33.5
K-profiles	1.96
GDHC + dynamicTreeCut	74.0
nlnet + multi level	20.0
nlnet + label propagation	19.9
Real data^$^ (3724 genes, 164 samples)	
ARACNE+ multi level	631.2
K-profiles	467.9
GDHC + dynamicTreeCut	1784.5
nlnet + multi level	301.9
nlnet + label propagation	314.0

* Computer system used: memory: 16GB 1600 MHz DDR3, CPU 2.5 GHz Intel Core i7, operating system: OS X EI Capitan 10.11.4.

^#^ RAM usage <400M by all methods.

^$^ RAM usage <500M by all methods.

## Conclusions

In this manuscript, we presented a network reconstruction method based on nonlinear associations. Linear associations are also utilized as a special case of the general association. The method is based on the distance measure DCOL, which enjoys high sensitivity and efficient computation. The method is effective in reconstruction of the network based on marginal nonlinear associations. The nonlinear network reconstruction method can also capture linear relations. However when sample size is not large, its statistical power to detect linear relations is lower than methods developed specifically for linear relations. Thus it may lose some weak linear relations, while gaining nonlinear relations. At the same time, currently there is no straight-forward definition of conditional independence based on DCOL. Thus such a nonlinear methods could be seen as a compliment to linear network reconstruction methods. It can potentially be combined with linear methods to make the results more comprehensive.

## References

[1] Patti GJ, Yanes O, Siuzdak G. Innovation: Metabolomics: the apogee of the omics trilogy. Nature reviews Molecular cell biology. 2012;13(4):263–9. 10.1038/nrm3314 22436749 PMC3682684

[2] Rung J, Brazma A. Reuse of public genome-wide gene expression data. Nature reviews Genetics. 2013;14(2):89–99. 10.1038/nrg3394 . 23269463

[3] Yu T, Bai Y. Network-based modular latent structure analysis. BMC bioinformatics. 2014;15 Suppl 13:S6 10.1186/1471-2105-15-S13-S6 25435002 PMC4248660

[4] Cho DY, Kim YA, Przytycka TM. Chapter 5: Network biology approach to complex diseases. PLoS Comput Biol. 2012;8(12):e1002820 10.1371/journal.pcbi.1002820 23300411 PMC3531284

[5] Thompson D, Regev A, Roy S. Comparative analysis of gene regulatory networks: from network reconstruction to evolution. Annu Rev Cell Dev Biol. 2015;31:399–428. 10.1146/annurev-cellbio-100913-012908 . 26355593

[6] Siegenthaler C, Gunawan R. Assessment of network inference methods: how to cope with an underdetermined problem. PloS one. 2014;9(3):e90481 10.1371/journal.pone.0090481 24603847 PMC3946176

[7] Langfelder P, Horvath S. WGCNA: an R package for weighted correlation network analysis. BMC bioinformatics. 2008;9:559 10.1186/1471-2105-9-559 19114008 PMC2631488

[8] Zhang B, Horvath S. A general framework for weighted gene co-expression network analysis. Stat Appl Genet Mol Biol. 2005;4:Article17 10.2202/1544-6115.1128 . 16646834

[9] Schafer J, Strimmer K. An empirical Bayes approach to inferring large-scale gene association networks. Bioinformatics. 2005;21(6):754–64. 10.1093/bioinformatics/bti062 . 15479708

[10] Peng J, Wang P, Zhou N, Zhu J. Partial Correlation Estimation by Joint Sparse Regression Models. J Am Stat Assoc. 2009;104(486):735–46. 10.1198/jasa.2009.0126 19881892 PMC2770199

[11] Chen X, Chen M, Ning K. BNArray: an R package for constructing gene regulatory networks from microarray data by using Bayesian network. Bioinformatics. 2006;22(23):2952–4. 10.1093/bioinformatics/btl491 . 17005537

[12] Vignes M, Vandel J, Allouche D, Ramadan-Alban N, Cierco-Ayrolles C, Schiex T, et al Gene regulatory network reconstruction using Bayesian networks, the Dantzig Selector, the Lasso and their meta-analysis. PloS one. 2011;6(12):e29165 10.1371/journal.pone.0029165 22216195 PMC3246469

[13] Villaverde AF, Ross J, Moran F, Banga JR. MIDER: network inference with mutual information distance and entropy reduction. PloS one. 2014;9(5):e96732 10.1371/journal.pone.0096732 24806471 PMC4013075

[14] Margolin AA, Nemenman I, Basso K, Wiggins C, Stolovitzky G, Dalla Favera R, et al ARACNE: an algorithm for the reconstruction of gene regulatory networks in a mammalian cellular context. BMC bioinformatics. 2006;7 Suppl 1:S7 10.1186/1471-2105-7-S1-S7 16723010 PMC1810318

[15] Liang KC, Wang X. Gene regulatory network reconstruction using conditional mutual information. EURASIP J Bioinform Syst Biol. 2008:253894 10.1155/2008/253894 18584050 PMC3171392

[16] Wang J, Chen B, Wang Y, Wang N, Garbey M, Tran-Son-Tay R, et al Reconstructing regulatory networks from the dynamic plasticity of gene expression by mutual information. Nucleic Acids Res. 2013;41(8):e97 10.1093/nar/gkt147 23470995 PMC3632132

[17] Li Y, Jackson SA. Gene Network Reconstruction by Integration of Prior Biological Knowledge. G3. 2015;5(6):1075–9. 10.1534/g3.115.018127 25823587 PMC4478538

[18] Ceci M, Pio G, Kuzmanovski V, Dzeroski S. Semi-Supervised Multi-View Learning for Gene Network Reconstruction. PloS one. 2015;10(12):e0144031 10.1371/journal.pone.0144031 26641091 PMC4671612

[19] Hase T, Ghosh S, Yamanaka R, Kitano H. Harnessing diversity towards the reconstructing of large scale gene regulatory networks. PLoS Comput Biol. 2013;9(11):e1003361 10.1371/journal.pcbi.1003361 24278007 PMC3836705

[20] Allen JD, Xie Y, Chen M, Girard L, Xiao G. Comparing statistical methods for constructing large scale gene networks. PloS one. 2012;7(1):e29348 10.1371/journal.pone.0029348 22272232 PMC3260142

[21] Sahoo D, Dill DL, Gentles AJ, Tibshirani R, Plevritis SK. Boolean implication networks derived from large scale, whole genome microarray datasets. Genome Biol. 2008;9(10):R157 10.1186/gb-2008-9-10-r157 18973690 PMC2760884

[22] Li KC. Genome-wide coexpression dynamics: theory and application. Proc Natl Acad Sci U S A. 2002;99(26):16875–80. 10.1073/pnas.252466999 12486219 PMC139237

[23] Boscolo R, Liao JC, Roychowdhury VP. An information theoretic exploratory method for learning patterns of conditional gene coexpression from microarray data. IEEE/ACM transactions on computational biology and bioinformatics / IEEE, ACM. 2008;5(1):15–24. 10.1109/TCBB.2007.1056 . 18245872

[24] Chen J, Xie J, Li H. A penalized likelihood approach for bivariate conditional normal models for dynamic co-expression analysis. Biometrics. 2011;67(1):299–308. 10.1111/j.1541-0420.2010.01413.x 20374241 PMC2902622

[25] Zhao J, Zhou Y, Zhang X, Chen L. Part mutual information for quantifying direct associations in networks. Proc Natl Acad Sci U S A. 2016 10.1073/pnas.1522586113 . 27092000 PMC4983806

[26] Yu T, Peng H. Hierarchical clustering of high-throughput expression data based on general dependences. IEEE/ACM transactions on computational biology and bioinformatics / IEEE, ACM. 2013;10(4):1080–5. 10.1109/TCBB.2013.99 24334400 PMC3905248

[27] Efron B, Tibshirani R. Empirical Bayes methods and false discovery rates for microarrays. Genet Epidemiol. 2002;23(1):70–86. ISI:000176697800006. 12112249 10.1002/gepi.1124

[28] Strimmer K. fdrtool: a versatile R package for estimating local and tail area-based false discovery rates. Bioinformatics. 2008;24(12):1461–2. 10.1093/bioinformatics/btn209 . 18441000

[29] Aubert J, Bar-Hen A, Daudin JJ, Robin S. Determination of the differentially expressed genes in microarray experiments using local FDR. BMC bioinformatics. 2004;5:125 10.1186/1471-2105-5-125 15350197 PMC520755

[30] Wagner GP, Pavlicev M, Cheverud JM. The road to modularity. Nat Rev Genet. 2007;8(12):921–31. Epub 2007/11/17. nrg2267 [pii] 10.1038/nrg2267 . 18007649

[31] Raghavan UN, Albert R, Kumara S. Near linear time algorithm to detect community structures in large-scale networks. Physical review E, Statistical, nonlinear, and soft matter physics. 2007;76(3 Pt 2):036106 10.1103/PhysRevE.76.036106 . 17930305

[32] Blondel VD, Guillaume JL, Lambiotte R, Lefebvre E. Fast unfolding of communities in large networks. Journal of Statistical Mechanics: Theory and Experiment. 2008;2008(10):P10008 (12 pp.). 10.1088/1742-5468/2008/10/P10008

[33] Wang K, Zhao Q, Lu J, Yu T. K-Profiles: A Nonlinear Clustering Method for Pattern Detection in High Dimensional Data. BioMed research international. 2015;2015:918954 10.1155/2015/918954 26339652 PMC4538770

[34] Hubert L, Arabie P. Comparing partitions. Journal of Classification. 2(1):193–218. 10.1007/bf01908075

[35] Burns JC, Kelly MC, Hoa M, Morell RJ, Kelley MW. Single-cell RNA-Seq resolves cellular complexity in sensory organs from the neonatal inner ear. Nature communications. 2015;6:8557 10.1038/ncomms9557 26469390 PMC4634134

[36] Falcon S, Gentleman R. Using GOstats to test gene lists for GO term association. Bioinformatics. 2007;23(2):257–8. 10.1093/bioinformatics/btl567 . 17098774

[37] Moayedi Y, Basch ML, Pacheco NL, Gao SS, Wang R, Harrison W, et al The candidate splicing factor Sfswap regulates growth and patterning of inner ear sensory organs. PLoS genetics. 2014;10(1):e1004055 10.1371/journal.pgen.1004055 24391519 PMC3879212

[38] Slowik AD, Bermingham-McDonogh O. Notch signaling in mammalian hair cell regeneration. Trends in developmental biology. 2013;7:73–89. 25328289 PMC4199338

[39] Qin S, Zou Y, Zhang CL. Cross-talk between KLF4 and STAT3 regulates axon regeneration. Nature communications. 2013;4:2633 10.1038/ncomms3633 24129709 PMC3867821

[40] Qin S, Zhang CL. Role of Kruppel-like factor 4 in neurogenesis and radial neuronal migration in the developing cerebral cortex. Molecular and cellular biology. 2012;32(21):4297–305. 10.1128/MCB.00838-12 22907754 PMC3486145

[41] Urness LD, Wang X, Shibata S, Ohyama T, Mansour SL. Fgf10 is required for specification of non-sensory regions of the cochlear epithelium. Developmental biology. 2015;400(1):59–71. 10.1016/j.ydbio.2015.01.015 25624266 PMC4361244

[42] Pauley S, Wright TJ, Pirvola U, Ornitz D, Beisel K, Fritzsch B. Expression and function of FGF10 in mammalian inner ear development. Developmental dynamics: an official publication of the American Association of Anatomists. 2003;227(2):203–15. 10.1002/dvdy.10297 12761848 PMC3904739

[43] Kiernan AE, Xu J, Gridley T. The Notch ligand JAG1 is required for sensory progenitor development in the mammalian inner ear. PLoS genetics. 2006;2(1):e4 10.1371/journal.pgen.0020004 16410827 PMC1326221

[44] Okano T, Xuan S, Kelley MW. Insulin-like growth factor signaling regulates the timing of sensory cell differentiation in the mouse cochlea. The Journal of neuroscience: the official journal of the Society for Neuroscience. 2011;31(49):18104–18. 10.1523/JNEUROSCI.3619-11.2011 . 22159122 PMC6634146

[45] Aburto MR, Magarinos M, Leon Y, Varela-Nieto I, Sanchez-Calderon H. AKT signaling mediates IGF-I survival actions on otic neural progenitors. PloS one. 2012;7(1):e30790 10.1371/journal.pone.0030790 22292041 PMC3264639

[46] Shpargel KB, Makishima T, Griffith AJ. Col11a1 and Col11a2 mRNA expression in the developing mouse cochlea: implications for the correlation of hearing loss phenotype with mutant type XI collagen genotype. Acta Otolaryngol. 2004;124(3):242–8. . 15141750 10.1080/00016480410016162

[47] Yan X, Lin J, Wang H, Markus A, Wree A, Rolfs A, et al Regional expression of the ADAMs in developing chicken cochlea. Developmental dynamics: an official publication of the American Association of Anatomists. 2010;239(8):2256–65. 10.1002/dvdy.22360 . 20658692

[48] Lin J, Yan X, Wang C, Talabattula VA, Guo Z, Rolfs A, et al Expression patterns of the ADAMs in early developing chicken cochlea. Development, growth & differentiation. 2013;55(3):368–76. 10.1111/dgd.12051 . 23496030

[49] Yamamoto N, Chang W, Kelley MW. Rbpj regulates development of prosensory cells in the mammalian inner ear. Developmental biology. 2011;353(2):367–79. 10.1016/j.ydbio.2011.03.016 . 21420948

